# Reliability of online pregnancy-related information and associated feelings of worry among expectant women in Qatar

**DOI:** 10.1186/s12884-022-04457-w

**Published:** 2022-02-11

**Authors:** Ayman Al-Dahshan, Mohamad Chehab, Noora Al-Kubaisi, Nagah Selim

**Affiliations:** 1 Department of Medical Education, Community Medicine Residency Program, Hamad Medical Corporation, P.O. Box 3050, Doha, Qatar; 2 Department of Clinical Affairs, Primary Health Care Corporation, Doha, Qatar; 3 Department of Public Health and Preventive Medicine, Faculty of Medicine, Cairo University, Cairo, Egypt

**Keywords:** Pregnancy, Health information, Online, Web-based, Worry, Reliability, Qatar

## Abstract

**Background:**

Although the internet can be a source of reassurance and clarification for expectant women, it could cause concerns or feelings of worry when reading about pregnancy-related information. This research study sought to assess feelings of worry and perceived reliability of online pregnancy-related information and the associated factors among expectant women attending antenatal clinics at primary healthcare centers in Qatar.

**Methods:**

A cross-sectional study design was used. The participants were recruited through a systematic random sampling technique. A self-administered questionnaire was used to collect data from the participants. Descriptive and analytic statistics were used as appropriate.

**Results:**

A total of 327 expectant women completed the questionnaire. Most were aged between 26–34 years (74.1%), held a college/university degree (76.4%), and were multigravidas (73.1%). About one-third of the women (31.2%) reported feeling worried due to information they read online. They coped with these feelings by consulting their antenatal care provider at their next appointment (51.0%) or by talking with relatives and friends (47.0%). Most participants (79.2%) considered online pregnancy-related information to be reliable or highly reliable. Holding a college/university degree and being primigravidae were factors significantly associated with a high perception of reliability of online pregnancy information.

**Conclusion:**

Although online pregnancy information caused feelings of worry for some expectant women, most perceived such information to be reliable. Thus, antenatal care providers should guide expectant women on how to access high-quality web-based information.

## Introduction

Pregnancy is a critical phase in any woman’s life. In addition to associated physical changes, expectant women experience a wide spectrum of lifestyle modifications and uncertainty throughout their pregnancy. To maintain some level of certainty, many seek information about the progression of their current pregnancy and the health of their fetus. They receive such information from a variety of sources such as their health care providers, family and friends, books, childbirth classes, and the internet [1].

Typically, antenatal clinical consultations are a major source of pregnancy-related information for expectant women. As they are high information-seekers, their demand for information cannot be fulfilled by the prolonged gap between antenatal care visits. Thus, they resort to the internet because of its convenience, ease of accessibility, and direct availability [2]. However, the wide scope and enormous quantity of online information can be tiresome to navigate and formidable among this cohort of women [3].

Being exposed to several sources may increase the possibility of receiving contradictory information. This is compounded by the fact that women surfing the internet for pregnancy-related information might lack the core skills for proper navigation and interpretation of these electronic health sources [4]. Despite its potential to transform the model of health care for patients and providers, there remains doubt about the quality and reliability of online information [5].

Nonetheless, several studies have shown that the majority of pregnant women consider the internet to be a highly reliable source of information. A study of Turkish pregnant women found that almost half (45%) had used the internet for health information. Upon rating the reliability of this information, the mean score was 7.13/10 [6]. Another survey of Italian pregnant women found that the majority (86%) had used the internet for pregnancy-related information [7]. Nearly two-thirds (64.1%) reported being highly confident about the reliability of the online information. As well, a study of pregnant Swedish women showed that the majority (84%) had used the internet for health information, and almost two-thirds had perceived the online information to be highly reliable [8].

Although the internet can be a source of reassurance and clarification for expectant women, it could also cause concerns or feelings of worry when reading about pregnancy-related information. A recent study conducted in Sweden found that almost two-thirds reported feelings of worry after retrieving pregnancy-related information on the internet [3]. Another study in Italy among women calling a hotline for teratology information showed that nearly one-third (30.2%) were alarmed by the online information they had found [9]. A larger percentage of the participants (40.5%) was confused about the pregnancy-related information they had sought [9].

In Qatar, the antenatal care (ANC) service of uncomplicated pregnancies is mainly provided through publicly funded primary health care (PHC) centers. These services are free of charge and include clinical assessment, screening, management, and health promotion activities. Expectant women receive care by primary care physicians and midwives through 8–10 ANC visits [10]. Most women in Qatar (78.7%) use the internet for health information [11]. Evidence, however, is scarce about how expectant women perceive the reliability of online pregnancy-related information, whether such information causes feelings of worry, and how they cope with such feelings. Thus, this research study sought to assess the feelings of worry and perceived reliability of online pregnancy information and their associated factors among expectant women attending ANC clinics at PHC centers in Qatar.

## Methods

### Study design and setting

The current study used an analytical cross-sectional design. It was conducted at ANC clinics of PHC centers in Qatar during four months in 2019. At the time of the study, there were 25 PHC centers, accredited by Accreditation Canada and distributed across three geographical regions (North, West, and Central) according to their respective population densities. These centers are considered the preferred first line of contact between the community and health care services and each serving populations of various ethnic, cultural, social and educational backgrounds [10]. Two health centers were randomly chosen from each region resulting in six health centers for inclusion in the study.

### Study population and sampling

The study population includes expectant women, aged ≥ 18 years, during any trimester of their pregnancy, speaking English or Arabic, and visiting the ANC clinic at one of the selected health centers during the study period. The participants were recruited through a systematic random sampling technique (every other woman). The sampling frame was constructed from the daily appointment list of the ANC clinics in each of the chosen health centers until the required sample size was reached.

Sample size.

According to the Planning and Statistics Authority in Qatar there were 468,510 women in childbearing ages (15 to 49 years) in 2018. Most women in Qatar (78.7%) use the internet to search for health information [11]. Thus, the estimated sample size for this study was calculated to be 323 individuals based on a 95% CI, precision of 5%, and a hypothesis that 70% (± 5%) of expectant women searched the internet for health-related information. The calculation of the sample size was performed to obtain a sufficiently precise estimate of the minimum number of study participants to ensure study power.

### Data collection

The data were collected through an anonymized, self-administered questionnaire. Trained research assistants, who were registered nurses and midwives, were situated in the chosen PHC centers to recruit and interview potential participants. They approached potential participants in the waiting areas of the ANC clinics. After informing them about the nature and purpose of the study, they invited them to participate with the knowledge that their participation was voluntary and declining participation or withdrawing from the study would have no effect on their quality of care. Those who agreed to participate were requested to sign a standardized informed consent form and were given the questionnaire to complete in their preferred language, English or Arabic.

### Questionnaire

A structured questionnaire was constructed after a comprehensive review of the literature. It consists of four main sections. The first section includes questions on socio-demographic characteristics (e.g., age, level of education, and occupation). The second section consists of questions regarding the characteristics of current pregnancy (e.g., parity, number of living children, gestational age, gender of the baby, health issues during current pregnancy). The third section contains questions on the participants’ feelings of worry from online pregnancy-related information. The fourth section is composed of questions regarding perceived reliability of online health information (e.g., extent of reliability, factors used in judging the reliability of online pregnancy-related information). The questionnaire was translated and back-translated (English-Arabic) by two independent translators. Any disagreements were addressed through discussion. The questionnaire was piloted among 15 pregnant women to obtain feedback on clarity and interpretation of questions and to estimate the time required to complete the survey. No modifications were necessary after the pilot phase.

### Statistical analysis

The collected data were analyzed using the Statistical Package for the Social Sciences (SPSS) version 23 (IBM Corp). Where appropriate, descriptive statistics for continuous and categorical variables were calculated. Pearson's χ2-test and Fisher’s exact test were used to assess the association between the outcomes and the independent variables. The level of statistical significance was set at 0.05.

## Results

### Demographic characteristics of respondents

A total of 327 expectant women completed the questionnaire with a response rate of 86% with time constraints being the main reasons for non-participation. Their characteristics are shown in Table 1. Most participants were between 26–34 years old (74.1%) and held a college/university degree (76.4%). Also, almost half (52.8%) of the pregnant women were in their second trimester and 69.4% had children at home. More than half (56.9%) did not discuss any information they found online with their healthcare provider.

**Table 1:** Background characteristics of the study participants (*N* = 327)

Variable	n (%)
Age (year)	
18–25	48 (14.8)
26–34	240 (74.1)
35 or more	36 (11.1)
Level of education	
Primary education	17 (5.2)
Secondary education	60 (18.4)
College/ University degree	249 (76.4)
Employment status	
Not working	197 (60.2)
Working	130 (39.8)
Parity	
Primigravida	88 (26.9)
Multigravida	239 (73.1)
Living children	
No	100 (30.6)
Yes	227 (69.4)
Trimester	
First	18 (5.6)
Second	171 (52.8)
Third	135 (41.7)
Gender of the fetus	
Male	121 (37.0)
Female	83 (25.4)
Unknown	123 (37.6)
Any health problems during current pregnancy	
No	76 (23.2)
Yes	251 (76.8)
Discuss online information with my healthcare provider	
No	182 (56.9)
Yes	138 (43.1)

### Feelings of worry caused by online pregnancy-related information

When asked about any feelings of worry due to online pregnancy-related information (Table 2), about one-third (31.2%) reported feeling worried after reading the online pregnancy-related information. Among those (*n* = 100), the two most frequent sources of worry were social media accounts (47%) and websites (38%). To cope with these feelings of worry, nearly half of the participants sought advice from their healthcare providers at the ANC clinic (51%) or through family and friends (47%).

**Table 2:** Feelings of worry caused by pregnancy-related information and coping methods among the participants (*N* = 327)

Variable	Agree, n (%)	Disagree, n (%)
Online pregnancy-related information makes me feel worried	100 (31.2)	221 (68.8)
Health information from the following online source(s) make me worried^a^ (*n* = 100)		
Social media	47 (47.0)	53 (53.0)
Internet websites	38 (38.0)	62 (62.0)
Forums	17 (17.0)	83 (83.0)
Mobile applications	10 (10.0)	90 (90.0)
I do the following to cope with feelings of worry (*n* = 100)		
Ask healthcare professional when visiting the ANC clinic	51 (51.0)	49 (49.0)
Ask family members or friends for support	47 (47.0)	53 (53.0)
Ask healthcare professional when visiting the general clinic	27 (27.0)	73 (73.0)
I do nothing	8 (8.0)	92 (92.0)

^a^multiple responses were allowed

### Perceived reliability of online pregnancy-related information

**Fig. 1:**
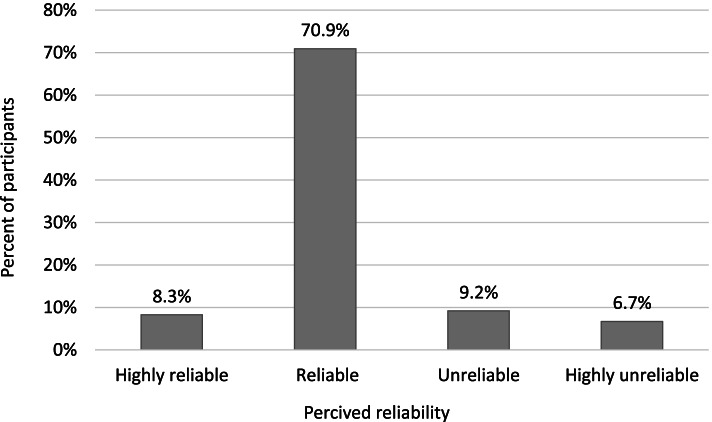
Participants’ perception of the reliability of online pregnancy-related information (*N* = 327)

Regarding the participants’ perceived reliability of online health information, 70.9% and 8.3% of the participants considered it to be reliable and highly reliable, respectively. The remaining participants considered it to be unreliable (9.2%) or highly unreliable (6.7%) (Fig. 1).

**Fig. 2:**
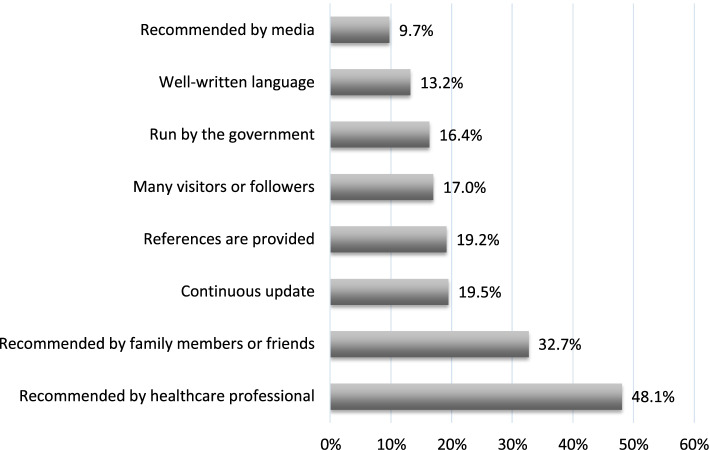
Factors to judge the reliability of online sources (*N* = 327)

To examine participants’ perceptions of the reliability of online health information further, they were asked to select three factors from a list of eight that defined how they judged the reliability of these online sources. The two most frequently reported factors were the recommendation of a healthcare professional (48.1%) and of a family member or friend (32.7%) (Fig. 2).

### The relationship between participants’ characteristics and perceived reliability of online pregnancy-related information

When the relationship between perceived reliability of online health information and the expectant mothers’ characteristics was examined using the chi-square test, there was a statistically significant association between participants’ perceived reliability and their level of education (*p* = 0.036) and gravidity (*p* = 0.006). Pregnant women with a college/university degree and those who were primigravidae were more likely to perceive the internet to be a reliable source of health information on pregnancy (Table 3).

**Table 3:** The relationship between participants’ characteristics and perceived reliability of online pregnancy-related information (*N* = 311)

Variable	Reliable, n (%)	Unreliable, n (%)	*p*-value
Age (year)			0.684
18–25	36 (81.8)	8 (18.2)	
26–34	195 (84.4)	36 (15.6)	
35 or more	26 (78.8)	7 (21.2)	
Level of education			0.036^a^
Up to secondary	56 (75.7)	18 (24.3)	
college/university	203 (86.0)	33 (14.0)	
Employment status			0.060
Not working	148 (80.0)	37 (20.0)	
Working	111 (88.1)	15 (11.9)	
Gravida			0.006^a^
Primigravida	78 (92.9)	6 (7.1)	
Multigravida	181 (79.7)	46 (20.3)	
Trimester			0.294
First	15 (83.3)	3 (16.7)	
Second	128 (80.0)	32 (20.0)	
Third	113 (86.9)	17 (13.1)	
Health problem during current pregnancy			0.076
No	51 (76.1)	16 (23.9)	
Yes	208 (85.2)	36 (14.8)	
Sharing online information with a healthcare provider			0.243
No	143 (81.3)	33 (18.8)	
Yes	113 (86.3)	18 (13.7)	

^a^Statistically significant result (*p* < 0.05)

Regarding the method of obtaining online pregnancy-related information (Table 4), those who used information websites were significantly more likely to perceive them as reliable sources compared to those who used other online sources including online forums, social media, and mobile applications.

**Table 4:** The relationship between mode of accessing online pregnancy-related information and perceived reliability of such information (*N* = 311)

Variable	Reliable n (%)	Unreliable n (%)	*p*-value
Information website			0.026^a^
No	75 (76.5)	23 (23.5)	
Yes	182 (86.7)	28 (13.3)	
Online forum			0.061^
No	232 (82.3)	50 (46.5)	
Yes	26 (96.3)	1 (3.7)	
Social media			0.503
No	154 (82.4)	33 (17.6)	
Yes	104 (85.2)	18 (14.8)	
Mobile application			0.777
No	131 (82.9)	27 (17.1)	
Yes	127 (84.1)	24 (15.9)	

^a^Statistically significant result (*p* < 0.05); ^Fisher’s exact test

## Discussion

The current study investigated the feelings of worry and perceived reliability of online pregnancy-related information among expectant women and their associated factors in Qatar. It was found that a large proportion of study participants reported not being worried about pregnancy information on the internet (68.8%) and considered such information to be reliable or highly reliable (79.2%). Accessing the internet during pregnancy is both a source of relief and worry simultaneously. While the internet may increase women’s knowledge about their health, it may trigger or amplify anxiety due to the vast amount of generic information with little or no clarification being offered [9].

Given that most women in Qatar (78.7%) use the internet for health information [11], it was vital to assess their feelings of worry associated with it. In our study, a large proportion (68.8%) of expectant women reported not being worried after the search for health information on the internet. On the other hand, a study among Swedish pregnant women revealed that many participants (65.6%) reported feelings of worry after reading online pregnancy-related information [3]. This difference might be explained by the higher educational level (76.4% vs 54.9% college level) in our sample.

Among expectant women, pregnancy can be a stressful phase of their life and might present their first major contact with the healthcare system. It involves several ANC clinical visits, laboratory investigations, and radiological exams. As a result, many women may prefer to seek reassurance through online resources. However, online sources may not always accommodate an individual’s threshold for fear, worry, and anxiety [12, 13]. Also, the search results might not be prioritized to reflect each user’s level of comprehension, clinical judgement, and health literacy. Although a large proportion of the participants in this study denied being worried when accessing online health information, health care professionals have a vital role in flagging to expectant women valid and reliable online resources that promote reassurance during their pregnancy [14].

Social media accounts (47%) and websites (38%) were the two most common triggers of worry among our participants. This was consistent with the findings in a Swedish study where websites (43.7%) and social media (25.7%) were among the most frequently identified sources of worry when browsing online pregnancy-related information [3]. This may be attributable to the fact that expectant women who post information on virtual platforms might not be inclined to share as much positive experiences as negative ones. Consequently, the negative aspects of pregnancies, including common physiological and pathological symptoms, may be inflated. To cope with these feelings of worry, almost half of the participants in the present study sought advice from their healthcare providers (51%) or family and friends (47%). Similarly, a survey of Turkish pregnant women found that nearly half (51%) had shared online health information with their healthcare providers [6]. In contrast, a systematic review on internet use among pregnant women reported that the majority refrained from discussing any information they retrieved online with their health care providers [15]. Thus, there is a need for antenatal care providers to acquire the necessary knowledge and skills to foresee and prevent such situations or identify them early during ANC visits and warn their patients of the potential risks of misinformation on the internet.

Regarding the perceived reliability of online health information, our finding of 79.2% participants reporting online information to be reliable or highly reliable is consistent with an earlier Chinese study in which most pregnant women (90.9%) found such information to be of medium–high reliability [16]. Also, a cross-sectional study among Italian pregnant women found that the majority (96.4%) had moderate-high confidence in online health information [17]. Another interesting finding in our study was that the participants’ level of education was significantly associated with their perceived reliability of online health information. It may be inferred that individuals with higher levels of education might possess better critical thinking and reasoning skills. However, a possible drawback of this might be that educated pregnant women may mistakenly consider themselves to be experts in judging the reliability of online health information [15]. Another factor that was significantly associated with perceived reliability of online information was the parity of the expectant woman. Primigravida women compared to multigravida were more likely to perceive online health information as reliable possibly because they are usually younger and have less fear of any age-related pregnancy or postpartum complications or congenital defects [18]. As well, they might be less likely to have gained a full spectrum of experience from conception until birth and parenthood. Thus, they might rely on the internet more to learn about what to anticipate during their current pregnancy and consider themselves to be capable of independently judging the reliability of online health resources. These findings reinforce the importance of equipping healthcare professionals with the knowledge and skills to provide tailored counselling for pregnant women in Qatar based on their characteristics, health literacy level, and information technology savviness.

The two most frequently reported factors for judging the reliability of online health resources by our study participants were the recommendation of a healthcare professional (48.1%) and a family member or friend (32.7%). In contrast, Turkish pregnant women attributed their perceived reliability of online health information to it being given by an expert (29.3%) and its frequency of usage (18.5%) [6]. While Chinese pregnant women judged the reliability of online health resources by cross-checking with other sources (67%), presence of references (42.1%), and validation by experts (34%) [16]. Currently, several guidelines and online tools have been developed to assist internet users in assessing the reliability of any information online [19, 20]. However, these instruments have several drawbacks such as placing a burden on internet users to check the accuracy of the information and on providers to ensure the currency and accuracy of the information (20). Thus, the implication for public health officials in Qatar will be to pursue the development of a locally adapted tool for assessing the reliability of online pregnancy information by actively engaging pregnant women and ANC health care professionals in the process.

Finally, the recent COVID-19 pandemic may have influenced pregnant women’s use of online pregnancy-related information and their perceptions about the reliability of these resources. As a result, COVID-19 pandemic brings opportunities and challenges for the development and promotion of reliable online antenatal resources.

### Strengths and limitations

This study possesses several strengths. Firstly, it was the first study to evaluate the feelings of worry and perceived reliability of online pregnancy-related information among expectant women in Qatar. Secondly, the study achieved a high response rate of 86%. Thirdly, a probability sampling technique was used to recruit participants in order to reduce the risk of sampling bias. Finally, while not being a population-based study, recruiting participants from PHC centers that serve populations of diverse backgrounds may offer a possible representation of expectant women in Qatar.

However, the present study was not without limitations. First, it was conducted among expectant women visiting the ANC clinics in publicly funded primary care settings. Hence, the results might not be generalizable to other expectant women seeking ANC services in the private health sector. Future studies should involve a larger sample of expectant women including those who visit private ANC providers. Also, it would be useful to collect additional data on socio-demographics, economic and educational backgrounds, as well as on health literacy to assess their relationships with reliability. Secondly, the cross-sectional design of our study was not enough to establish a causal relationship between perceived reliability and participants’ characteristics. Thirdly, this study relied on self-report, which may be subject to recall and social desirability biases.

## Conclusion

Although online pregnancy information caused feelings of worry for some expectant women in this study, most perceived such information to be reliable. Holding a college/university degree and being primigravida were factors significantly associated with a high perception of reliability of online pregnancy-related information. Several areas for future action by public health officials have been uncovered. There is a solid need for evaluating the reliability and accuracy of the most frequently used online sources on pregnancy-related issues. In addition, antenatal care providers should be equipped to guide pregnant women on how to access high-quality web-based information. Also, they should assume the responsibility of discussing such information with their patients during their antenatal visits.

## Data Availability

The datasets used and/or analyzed during the current study are available from the corresponding author on reasonable request.
